# Mesenteric panniculitis: A case report

**DOI:** 10.1016/j.ijscr.2025.111000

**Published:** 2025-02-01

**Authors:** Rabti Souphia, Ben Marzouk Sawssen, Farjaoui Wael, Mohamed Hedi Mannai, Khalifa Mohamed Bechir

**Affiliations:** General Surgery Department, Military Hospital of Tunis, Mont Fleury-1008, Tunis, Tunisia; Faculty of Medicine of Tunis, 15, Djebel Lakhdhar Street – 1007 Bab Saadoun, Tunis, Tunisia

**Keywords:** Mesenteric panniculitis, Retractile mesenteritis, Abdominal CT scan, Chronic inflammation, Adipose tissue fibrosis

## Abstract

**Introduction and importance:**

Mesenteric panniculitis is a rare, benign condition characterized by chronic inflammation and fibrosis of mesenteric adipose tissue (Hussein and Abdelwahed, 2015; Gögebakan et al., 2018 [1, 2]). While its etiology remains unclear in many cases, it has been associated with various conditions including abdominal surgery, trauma, and inflammatory diseases (Buragina et al., 2019 [3]). Understanding its presentation and management is crucial for proper patient care.

**Case presentation:**

We present a case of mesenteric panniculitis in a 49-year-old woman who presented with significant weight loss. The diagnosis was confirmed through imaging studies and tissue biopsy, with successful symptomatic management through conservative treatment.

**Clinical discussion:**

The condition typically presents with nonspecific symptoms including abdominal pain, nausea, and weight loss, though it may be discovered incidentally. Diagnosis relies on a combination of imaging findings, particularly the characteristic “misty mesentery” appearance on CT scan, and when indicated, histological confirmation.

**Conclusion:**

Early recognition and appropriate management of mesenteric panniculitis are essential for optimal outcomes. While usually benign, the condition requires thorough evaluation to exclude underlying malignancy and careful monitoring to prevent complications.

## Highlights

• Mesenteric panniculitis is a rare and benign condition characterized by chronic inflammation and fibrosis of the mesenteric fat.
• The disease progresses through three stages, with the final stage presenting as retractile mesenteritis, potentially causing obstructive symptoms.
• CT imaging is crucial for diagnosis, often detecting mesenteric panniculitis incidentally.
• Treatment ranges from medical therapy to surgery, with follow-up imaging needed to check for malignancy.

## 1 Introduction

Mesenteric panniculitis represents a rare inflammatory condition affecting the adipose tissue of the mesentery [1,2]. The condition progresses through three distinct stages: lipodystrophy, panniculitis, and finally retractile mesenteritis [4]. Its prevalence remains uncertain, though studies suggest it affects approximately 0.6–2.5 % of patients undergoing abdominal CT scans [5].

The etiology remains poorly understood, with various proposed mechanisms including autoimmune responses, previous abdominal surgery, trauma, and underlying malignancy [3,6]. Recent studies have highlighted associations with IgG4-related disease and various inflammatory conditions [7,8]. Understanding this condition is crucial due to its potential impact on patient quality of life and its association with other underlying pathologies.

This work has been reported in line with the SCARE criteria [9].

## 2 Case report

### 2.1 Patient information

A 49-year-old woman presented with a 15-kg weight loss over six months. Her initial BMI was 28.4 kg/m^2^, decreasing to 22.7 kg/m^2^ at presentation. The patient has a medical history of controlled arterial hypertension, managed with a daily dose of amlodipine 5 mg. Surgical interventions include a McBurney appendectomy performed at the age of eight, an epigastric hernia repair with mesh placement in 2016, and an abdominoplasty in 2020 to address post-pregnancy abdominal wall laxity. Additionally, she underwent a laparoscopic cholecystectomy in 2022 due to symptomatic cholelithiasis.

**Figs. 1, 2:**
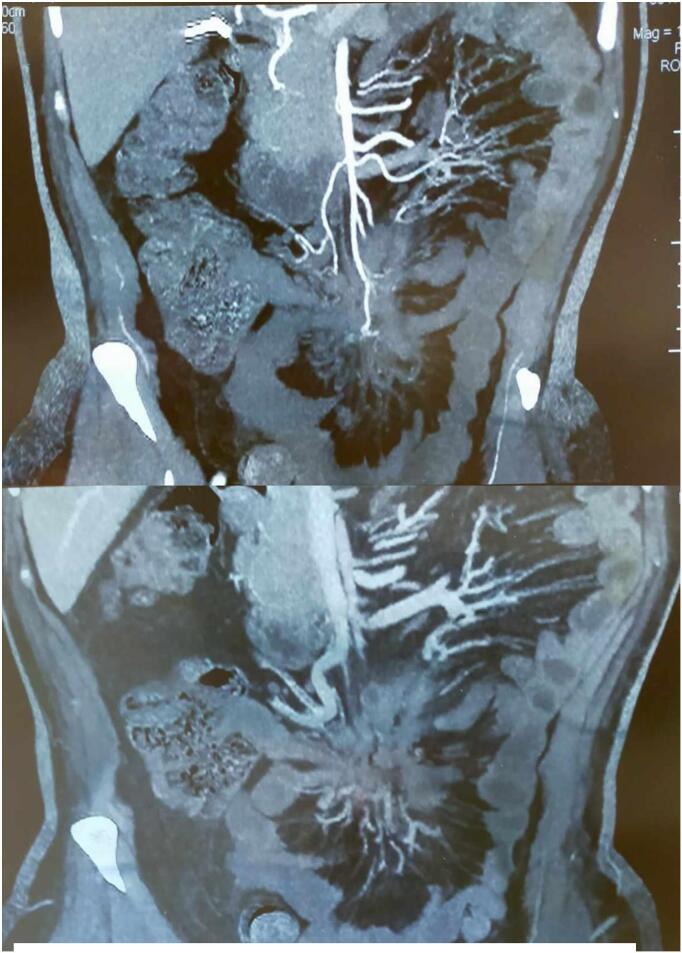
Scans of mesenteric panniculitis.

On physical examination, the patient had stable vital signs, with a blood pressure of 125/75 mmHg, a heart rate of 76 beats per minute, and a temperature of 36.8 °C. Abdominal examination revealed a soft abdomen with mild tenderness in the periumbilical region, without palpable masses, and well-healed surgical scars. Laboratory investigations showed a white blood cell count of 7.2 × 10^9^/L, hemoglobin at 13.4 g/dL, and platelets at 245 × 10^9^/L. The comprehensive metabolic panel, thyroid function tests, serum calcium, and phosphate levels were within normal limits, while inflammatory markers indicated an ESR of 22 mm/h and a CRP of 8 mg/L. Imaging studies, including an abdominal CT with contrast, revealed mesenteric panniculitis characterized by a “misty mesentery” appearance and mesenteric adenopathy (Figs. 1, 2). A colonoscopy was performed to rule out underlying inflammatory bowel disease or malignancy, with normal findings.

The patient was initially treated with analgesics (acetaminophen 1 g TID) and low-dose prednisone (20 mg daily with gradual taper), alongside regular monitoring of symptoms and inflammatory markers. At the three-month follow-up, there was a resolution of abdominal pain, stabilization of weight, and improvement in inflammatory markers. A six-month follow-up CT showed stable mesenteric changes, and the patient continues to undergo annual monitoring to assess for disease progression or the development of associated conditions.

## 3 Discussion

Mesenteric panniculitis presents a diagnostic challenge due to its nonspecific symptoms and varying clinical course. First described by Jura in 1924, the condition predominantly affects males with a ratio of 2–3:1 [10]. Understanding of its pathogenesis has evolved, with recent literature suggesting a complex interplay between inflammatory mediators and immune response [11].

The progression of the disease occurs in three stages: lipodystrophy, characterized by fat cell necrosis; panniculitis, marked by an inflammatory infiltrate; and retractile mesenteritis, distinguished by fibrosis and tissue retraction. Imaging plays a crucial role in diagnosis, with CT being the preferred modality, revealing characteristic findings such as a “misty mesentery” appearance, the fat ring sign, pseudocapsule formation, and lymph node involvement. While MRI can provide complementary information, particularly in cases where CT findings are inconclusive, it is considered an adjunct diagnostic tool rather than a second-line option [12]. Management strategies depend on symptom severity and the underlying cause, with conservative treatment using anti-inflammatory medications often providing adequate symptom control. However, surgical intervention is reserved for complications such as bowel obstruction or perforation [13].

## 4 Conclusion

Mesenteric panniculitis requires a high index of suspicion for diagnosis. While often benign, thorough evaluation is essential to exclude underlying malignancy. Regular monitoring and appropriate symptomatic management typically lead to favorable outcomes. This case highlights the importance of systematic evaluation and long-term follow-up in patients with mesenteric panniculitis.

## Author contribution

Rabti Souphia and Ben Marzouk Sawssen contributed to manuscript writing and editing, and data collection; Wael Farjaoui contributed to data analysis; Mohamed Hedi Mannai and Med Bachir Khalifa contributed to conceptualization and supervision; All authors have read and approved the final manuscript.

## Patient consent

Written informed consent was obtained from the patient for the publication of this case report and its accompanying images. A copy of the written consent is available for the Editor-in-Chief of this journal to review upon request.

## Ethical approval

Ethical approval is not applicable/waived at our institution. Due to the specific nature of case reports, which involve detailed descriptions of observations and interventions that have already been conducted on patients, as opposed to prospective studies involving planned interventions, our institution does not require formal ethical approval for such cases. We recognize the importance of ethics in medical research and are fully committed to upholding ethical standards in our medical and research practices.

## Guarantor

Rabti Souphia.

## Guarantor

Rabti Souphia.

## Research registration number

N/A.

## Funding

This research did not receive funding from any specific grant provided by public, commercial, or not-for-profit organizations.

## Conflict of interest statement

No conflicts of interest.
